# From early symptoms to EEG silence: tracking the neurodegenerative course of sporadic Creutzfeldt-Jakob disease

**DOI:** 10.3389/fnhum.2025.1652773

**Published:** 2025-10-28

**Authors:** Yunfang He, Liangliang Qiu

**Affiliations:** ^1^Department of Neurology, The First Affiliated Hospital of Fujian Medical University, Fuzhou, China; ^2^Department of Neurology, National Regional Medical Center, Binhai Campus of the First Affiliated Hospital, Fujian Medical University, Fuzhou, China

**Keywords:** Creutzfeldt-Jakob disease (CJD), electroencephalogram (EEG), periodic sharp wave complexes (PSWC), MRI, diffusion-weighted imaging (DWI), fluid-attenuated inversion recovery (FLAIR)

## Abstract

**Background:**

Sporadic Creutzfeldt-Jakob disease (sCJD) is a rapidly progressive and fatal neurodegenerative disorder. Early diagnosis remains challenging due to nonspecific initial symptoms. Although electroencephalography (EEG) is a key diagnostic tool, particularly through the detection of periodic sharp wave complexes (PSWCs), the longitudinal evolution of EEG features and their correlation with clinical and neuroimaging progression are not fully characterized.

**Methods:**

This retrospective cohort study analyzed 37 patients diagnosed with probable and very probable sCJD according to the 2021 Chinese diagnostic guidelines. All patients underwent at least one EEG examination. One representative patient was followed for 23 weeks with serial EEG and MRI studies to document dynamic electrophysiological and structural changes. EEG background activity was graded as mild, moderate, or severe, and PSWCs were identified based on standardized criteria. MRI analyses focused on the spatial and temporal progression of hyperintense lesions on DWI and FLAIR sequences.

**Results:**

Among the 37 patients, 46% underwent initial EEG within 1 month of symptom onset, and 49% exhibited severe background abnormalities. PSWCs were present in 84% of patients at first EEG, with a high prevalence (82%) even in those examined within 4 weeks of onset. Longitudinal analysis in the index case revealed a progressive EEG deterioration: from slowed and disorganized background rhythms and emerging triphasic waves at 8 weeks, to widespread PSWCs with increasing periodicity (9–16 weeks), and finally to a burst-suppression pattern near electrical silence by week 23. Concurrent MRI showed a parallel expansion of hyperintense lesions from unilateral cortical and basal ganglia regions to bilateral involvement, closely correlated with the EEG progression. Statistical analysis showed no significant correlation between survival time and age, time to first EEG, CSF 14–3-3 protein status, or initial EEG background grade. Furthermore, neither the presence of typical PSWCs nor the severity of background activity was associated with survival outcomes.

**Conclusion:**

EEG, especially the early and highly prevalent presence of PSWCs, offers high diagnostic value in sCJD but does not serve as prognostic predictors. The close correlation between EEG decline and MRI progression supports multimodal monitoring. Serial EEG should be integrated into sCJD diagnosis and follow-up.

## 1 Introduction

Creutzfeldt-Jakob disease (CJD) is a fatal neurodegenerative disorder caused by the cerebral deposition of abnormally folded host-encoded prion protein (Zerr et al., 2024). Human prion diseases can manifest in various phenotypes or subtypes, which may be familial, sporadic, or acquired. Sporadic CJD (sCJD) is the most common subtype of CJD, accounting for 85% of CJD cases, with an annual mortality rate of 1.39 per million people. sCJD is a rapidly progressing disease with an average survival of 6 months, and over 90% of patients die within 1 year of symptom onset (Hill et al., 2003). However, sCJD diagnosis is often delayed or missed because early manifestations of sCJD are non-specific and overlap with other rapidly progressive dementias (Pérez et al., 2024). Thus, there is essential to establish an early and reliable diagnostic framework before irreversible neuronal damage occurring, which is not only beneficial to exclude treatable encephalopathies but also beneficial to allow timely palliative care (Gurram et al., 2023; Jurcau et al., 2024).

For decades, electroencephalography (EEG) has been a key ancillary test for CJD diagnosis due to non-invasive, repeatable, and bedside operation (Huang et al., 2024), though, EEG in the early stage of the disease is often non-specific (O'Leary, 2022). Approximately 66–70% of CJD patients develop specific periodic sharp-wave complexes (PSWCs) 3 months after disease onset (Idowu et al., 2025). PSWC was included in the diagnostic classification criteria for CJD by the World Health Organization (1998). As for EEG, waves were conventionally divided by frequency into *δ* (0.5–3 Hz), *θ* (4–7 Hz), *α* (8–13 Hz), *β* (13–34 Hz), and *γ* (>35 Hz) bands, and by morphology into mono-, bi-, triphasic and complex waveforms. PSWCs are classically bilateral, synchronous triphasic sharp waves lasting 10–600 ms, recurring every 500–2000 ms in runs of ≥ 5 cycles with an inter-discharge jitter < 500 ms (Pierre, 1980). As cortical spongiform degeneration advances, PSWCs wane and are eventually replaced by electrical silence or persistent low-voltage activity. A 2024 multicenter study, including 312 autopsy-confirmed sCJD cases, reported a PSWC detection rate of 74% which was a positive predictive value for CJD of 95%.

Although the diagnostic value of EEG is recognized, most prior work has relied on single or short-duration cross-sectional recordings, leaving the longitudinal EEG trajectory from prodromal stages to end-stage disease largely unexplored (Hung et al., 2010). Moreover, the relationships among background-activity grade and the timing, duration and disappearance of PSWCs, and their predictive value for clinical outcomes, have not been systematically elucidated (Feng et al., 2021). These knowledge gaps limit the timeliness and precision of EEG in clinical decision-making.

Therefore, we performed this retrospectively and observational cohort study including 37 patients fulfilling “probable” or “very probable” sCJD criteria to analysis clinical data and EEG characteristics. In addition, a longitudinal following-up lasting 23 weeks was performed in one representative sCJD case, for detailed mapping the emergence, evolution, and extinction of PSWCs in EEG, and analyzing the correlation between EEG changes and clinical progression and MRI findings.

## 2 Methods

### 2.1 Study design and subjects

The study included 37 patients who were diagnosed as probable or very probable CJD according to the “Creutzfeldt-Jakob Disease Chinese Diagnosis Guidelines 2021” (Supplementary method) and were hospitalized in the Department of Neurology of the First Affiliated Hospital of Fujian Medical University between 2020 and 2024. Each patient received at least one EEG examination during their hospital stay. All cases were sporadic without a familiar history, organ transplantation, or biological agent treatment history. Informed consent was waived for this study by the Ethics Committee of the First Affiliated Hospital of Fujian Medical University (Approval No.: MTCA, ECFAH of FMU [2015] 084–2), because it is a retrospective, observational study using exclusively de-identified clinical data extracted from the hospital’s electronic medical record system.

### 2.2 EEG examination

EEG was recorded using a Natus Nicolet Monitor system (Natus Medical Incorporated, USA). A full set of 32 scalp electrodes was positioned according to the international 10–20 system. Electrode impedance was maintained below 5 kΩ throughout the recordings. The signals were sampled at 250 Hz and filtered using a band-pass filter of 0.5–70 Hz, with a 50 Hz notch filter applied to suppress line noise. All recordings included at least 30 min of resting-state data under awake conditions.

Routine unipolar and bipolar lead tracings were conducted for a minimum of 6 h. Provocation maneuvers (hyperventilation, photic stimulation) were performed when clinically feasible. Artifact rejection was performed manually during visual analysis. All EEGs were visually analyzed independently by two experienced neurologists (Y.H. and L.Q.), for background rhythm, reactivity, presence of PSWC, and focal slowing. Disagreements were resolved by consensus. PSWC were mainly defined as bilateral, synchronous triphasic sharp waves. The abnormal EEG background activity was graded as mild, moderate, and severe. Specifically, mild abnormalities were characterized by mixed slow-wave activity and normal *α* waves, with the overall pattern maintaining a certain level of organization and reactivity; moderate abnormalities presented as fragmented or completely absent background rhythms, dominated by *θ* and *δ* waves with a significant reduction in α waves; severe abnormalities were featured by highly disorganized and discontinuous activity, typically without reactivity or obvious EEG structure, mainly consisting of low-amplitude δ wave activity; extremely sever abnormalities were exhibited a completely disorganized and suppressed pattern, characterized by persistent low-voltage delta activity (often <20 μV), absence of reactivity, and eventual evolution into burst-suppression or near-electrical silence (often <2 μV). In short, the basic rhythm was defined as severe when below 4 Hz, moderate when between 4 and 7 Hz, and mild when above 7 Hz. Burst-suppression was defined as a pattern of intermittent bursts of sharp- and slow-wave complexes superimposed on a suppressed background (often <20 μV), whereas near-electrical silence was characterized by persistent, ultra-low voltage delta activity (<2 μV) without reactivity or discernible bursts (Liu, 2017).

### 2.3 Imaging examination

MRI examinations, including sequences of diffusion-weighted imaging (DWI) and fluid-attenuated inversion recovery (FLAIR), were performed using a 3.0 T MRI system (e.g., Siemens Prisma). The parameters for the DWI sequence were as follows: repetition time (TR) = 8,000 ms, echo time (TE) = 89 ms, b values = 0 and 1,000 s/mm^2^, slice thickness = 2 mm, and no slice gap. For the FLAIR sequence, the parameters were: TR = 8,000 ms, TE = 90 ms, inversion time (TI) = 2,500 ms, and slice thickness = 2 mm. The analysis process involved qualitatively two neuroradiologists, who were blinded to the clinical data, independently reviewing the images. They focused on observing the location, range, and dynamic changes of hyperintense lesions in the DWI and FLAIR sequences. By comparing images from different follow-up time points, they determined whether the lesions spread from a localized area to bilateral regions (e.g., from the left caudate nucleus and occipitoparietal cortex to bilateral caudate nuclei, lentiform nuclei, frontal lobes, insula, etc.). They also analyzed the consistency between these changes and abnormal electroencephalogram findings. Finally, the progression of the lesions was confirmed through consensus to reflect the degree of neuronal damage and prion protein deposition during the disease course.

### 2.4 Statistical analysis

Numerical data and categorical data were described with medians (ranges) and numbers (frequencies), respectively. The Mann–Whitney *U* test or Kruskal-Wallis *H* test was used to compare nonparametric variables; the χ^2^ test or Fisher’s exact test was used to compare categorical variables; the Spearman’s rank correlation test was used analyze correlations between survival time and age and time of first EEG examination post-onset. The Kaplan–Meier (K-M) curve was utilized to depict survival curves, and the log-rank test was employed to assess differences among patients with different grade of EEG background activities. Statistical analysis was performed with SPSS V.25.0. A 2-sided *p* < 0.05 was considered statistically significant.

## 3 Results

### 3.1 EEG characteristics and clinical manifestations of the CJD cohort

**Figure 1:**
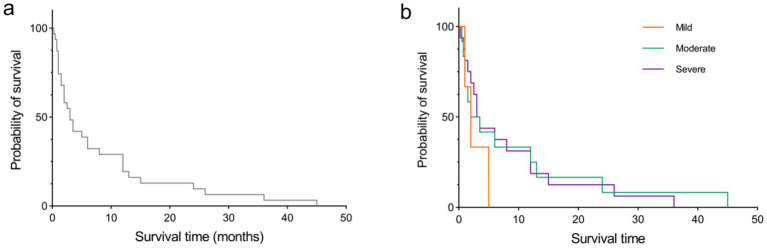
K-M curve of survival time. **(a)** for all sCJD patients in this study. **(b)** for the sCJD patients with difference background activities of mild, moderate, and sever at first time EEG examination.

Among the 37 sCJD patients, there were 17 males (46%) and 20 females (54%). The median age was 64 years (range: 33–90). Among them, only 4 patients (11%) were under 50 years old, and only 3 patients (8%) were over 80 years old. The median time for the first EEG examination among the 37 patients was 6 weeks after disease onset (range: 1 week to 40 weeks). Seventeen patients (46%) visited within 4 weeks (1 month) post of onset, and 6 patients (16%) visited more than 12 weeks (3 months) after onset. The initial symptoms included memory impairment in 10 patients (27.0%), movement disorders in 8 patients (22%), sluggishness in 7 patients (19%), dizziness in 6 patients (16%), speech disorders in 4 patients (11%), psychiatric behavioral abnormalities in 1 patient (3%), and visual disturbances in 1 patient (3%). The background activity grades of the first EEG examination were: mild in 4 patients (10.8%), moderate in 15 patients (41%), and severe in 18 patients (49%). The main discharge areas of the EEG were in the frontal lobe in 17 patients (46%), central-and-parietal areas in 4 patients (11%), frontal-central-and-parietal areas in 1 patient (3%), parietal area in 1 patient (3%), occipital area in 3 patients (8%), parietal-and-temporal areas in 1 patient (3%), frontal-and-temporal areas in 2 patients (5%), and temporal area in 2 patients (5.4%). Full lead discharge was found in 6 patients (16.2%) during the first EEG examination. 31 patients (84%) showed typical PSWC on first EEG examination. 24 of 30 patients (80%) showed positive in 14-3-3 protein of CSF. Only 1 of 34 patients (3%) alive at the time of this study performed. Median survival time of the 31 patients died with available survival time was 3 months (range: 0.25 to 45). K-M curve of survival time of this sCJD cohort was visualize in Figure 1a. Table 1 summarizes the clinical manifestations and EEG characteristics of the 37 CJD patients. Individual information was detailed in Table 2.

**Table 1:** EEG characteristics and clinical manifestations of 37 patients with sCJD.

Characteristics	Median (range) or *n* (%)
Total	37
Demographics	
Female, sex	20 (54.1)
Age, years	64 (33, 90)
First time of EEG examination post onset	
≤4 weeks	17 (45.9)
4 week<~ ≤ 12 weeks	14 (37.8)
> 12 weeks	6 (16.2)
Background activity grades of first time of EEG examination	
Mild	4 (10.8)
Moderate	15 (40.5)
Severe	18 (48.6)
Main EEG discharge area	
Frontal	17 (45.9)
Central-Parietal	4 (10.8)
Frontal-Central-Parietal	1 (2.7)
Parietal	1 (2.7)
Occipital	3 (8.1)
Parietal–Temporal	1 (2.7)
Frontal-Temporal	2 (5.4)
Temporal	2 (5.4)
Full-Lead	6 (16.2)
Typical PSWC on EEG	31 (83.8)
14-3-3 protein of CSF, n of positive/total n	24/30 (80%)
Survival, n/total n	1/34 (2.9%)
Survival time, months	3 (0.25, 45)

Percentages may not total 100 because of rounding. EEG, Electroencephalography; PSWC, periodic sharp wave complexes; sCJD, sporadic Creutzfeldt-Jakob disease.

**Table 2:** Individual EEG characteristics and clinical manifestations of 37 patients with sCJD.

No.	Gender	Age (year)	Time of first EEG examination post-onset (weeks)	Initial symptom	Basic rhythm of EEG (Hz)	Background activity grades	PSWC	Main discharge area (general classification)	1,433 protein of CSF	Survival time (months)
1	Female	66	1	Speech disorder	4–7	Moderate	Typical	Frontal area	Positive	NA
2	Male	45	2	Sluggishness	8.5–10	Mild	Atypical	Frontal area	Positive	2
3	Male	64	40	Memory impairment	2–6	Severe	Typical	Central-Parietal area	NA	1
4	Female	60	32	Memory impairment	2–6	Severe	Typical	Frontal area	NA	NA
5	Male	57	24	Memory impairment	2–6	Severe	Typical	Frontal area	NA	12
6	Female	57	4	Motor disorder	1.5–3	Severe	Typical	Frontal-Central-Parietal area	Positive	2
7	Female	58	1.5	Motor disorder	1.5–3.5	Severe	Typical	Parietal area	Positive	6
8	Female	57	3	Motor disorder	2–6	Severe	Typical	Frontal area	Positive	12
9	Female	69	8	Sluggishness	4–7	Severe	Typical	Occipital area	Negative	1.5
10	Male	51	12	Motor disorder	4–7	Moderate	Typical	Central-Parietal area	NA	0.5
11	Female	60	3	Motor disorder	8–11	Mild	Atypical	Frontal area	NA	1
	Male	66	12	Sluggishness	4–7	Moderate	Typical	Temporal area	Positive	45
13	Female	64	3	Psychotic behavioral abnormalities	1–3.5	Severe	Typical	Frontal area	Positive	15
14	Male	33	8	Speech disorder	1–3.5	Severe	Typical	Frontal area	Positive	36
15	Female	54	2.5	Memory impairment	1.5–3.5	Severe	Typical	Frontal area	Negative	3
16	Male	68	12	Sluggishness	4–7	Moderate	Typical	Full lead	Positive	1
17	Male	83	6	Dizziness	3–7	Moderate	Typical	Central-Parietal area	Negative	13
18	Male	67	4	Sluggishness	4–7	Severe	Typical	Full lead	Positive	2.5
19	Female	69	2	Speech disorder	4–7	Moderate	Typical	Full lead	Negative	6
20	Male	58	4	Dizziness	1.5–7	Moderate	Typical	Frontal area	Positive	24
21	Male	54	8	Memory impairment	4–7	Moderate	Typical	Frontal area	Positive	alive
22	Male	60	1	Memory impairment	4–7	Moderate	Typical	Frontal area	Positive	1.5
23	Male	80	1	Sluggishness	1.5–3.5	Severe	Typical	Frontal area	Positive	0.75
24	Female	49	4	Visual disturbance	4–7	Moderate	Typical	Occipital area	Positive	2
25	Female	39	8	Dizziness	4–7	Moderate	Typical	Frontal area	Positive	3.5
26	Female	68	8	Speech disorder	1.5–7	Moderate	Atypical	Temporal area	Positive	12
27	Male	72	28	Sluggishness	6.5–8.5	Mild	Atypical	Frontal-Temporal area	Negative	5
28	Female	70	6	Dizziness	1.5–3.5	Severe	Atypical	Occipital area	Positive	0.25
29	Female	69	3	Memory impairment	4–7	Severe	Typical	Parietal–Temporal area	Positive	died^a^
30	Male	90	24	Dizziness	7–9	Mild	Typical	Frontal-Temporal area	NA	died^a^
31	Female	58	4	Memory impairment	4–7	Moderate	Typical	Full lead	NA	NA
32	Female	69	6	Dizziness	4–7	Moderate	Typical	Frontal area	Positive	0.75
33	Male	75	6	Memory impairment	1.5–3.5	Severe	Typical	Full lead	Positive	8
34	Female	64	24	Motor disorder	4–7	Moderate	Typical	Frontal area	Positive	1
35	Female	58	6	Motor disorder	1.5–5	Severe	Typical	Central-Parietal area	Positive	26
36	Male	77	4	Motor disorder	1.5–3.5	Severe	Atypical	Frontal area	Positive	3.5
37	Female	75	8	Memory impairment	2.5–4	Severe	Typical	Full lead	Negative	3

^a^Death time was none available.

EEG, Electroencephalography; NA, none available; PSWC, periodic sharp wave complexes; sCJD, sporadic Creutzfeldt-Jakob disease.

### 3.2 Longitudinal EEG and MRI findings in a representative sCJD patient

**Figure 2:**
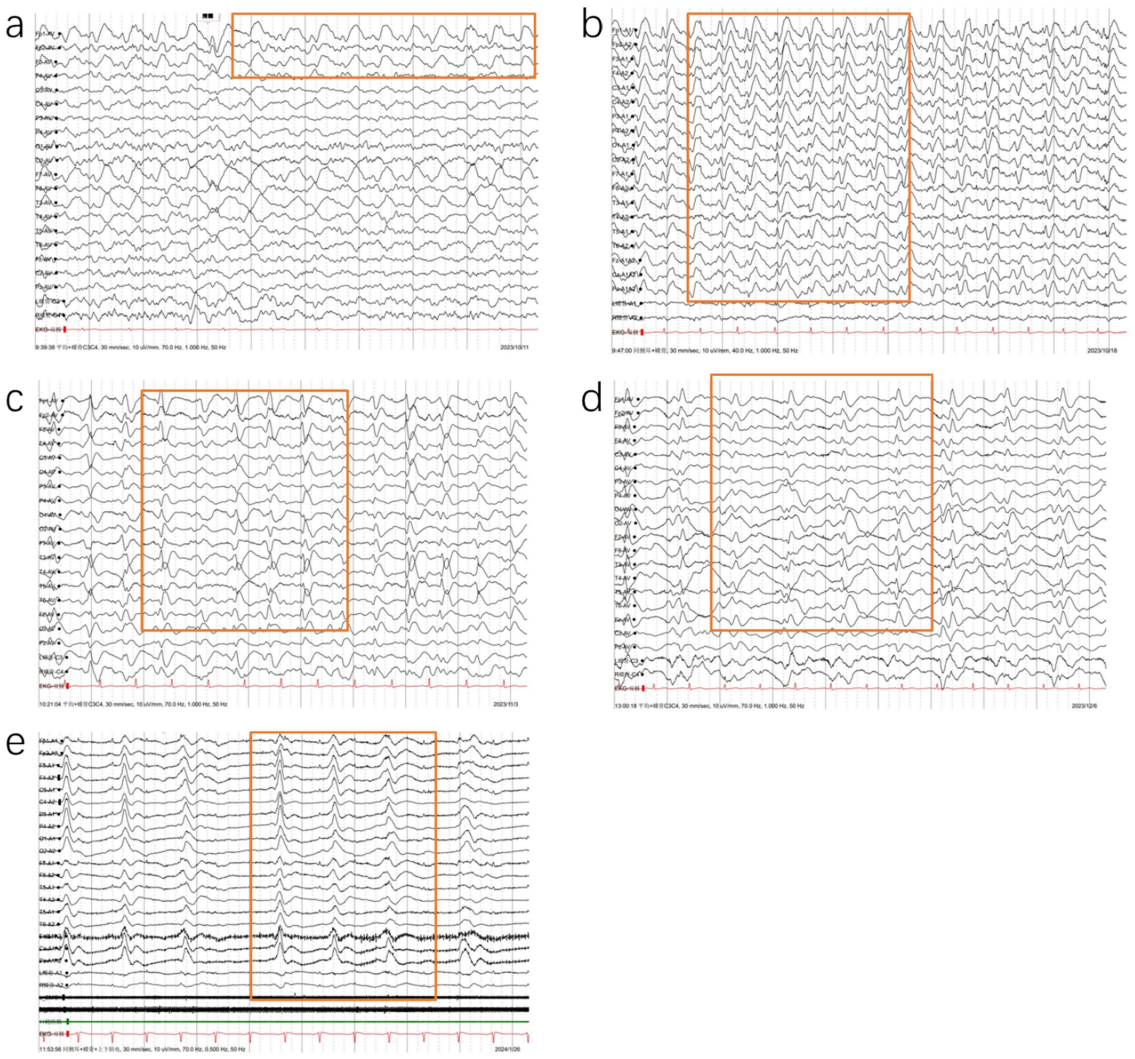
Follow-up of EEG changes in a patient with CJD: **(a)** Initial EEG (8 weeks post-onset) showing background disorganization with occipital *α* waves, fragmented rhythm, progressive 3–6 Hz slow waves, and emerging sporadic triphasic waves in the left frontotemporal region; **(b)** EEG at 9 weeks post-onset: disintegrated background with widespread 2–7 Hz slow waves and bilateral synchronous periodic triphasic waves at 0.4–0.6 s intervals; **(c)** EEG at 11 weeks post-onset (comatose state): further slowing of background (1.5–4 Hz), with prolonged (0.8–1.4 s) and rounded triphasic waves; **(d)** EEG at 16 weeks post-onset: low-voltage background with 1.5–3 Hz slow waves and periodic triphasic complexes at 1.0–1.5 s intervals; **(e)** EEG at 23 weeks post-onset (coma): burst-suppression pattern with intermittent sharp- and slow-wave complexes on a suppressed background.

**Figure 3:**
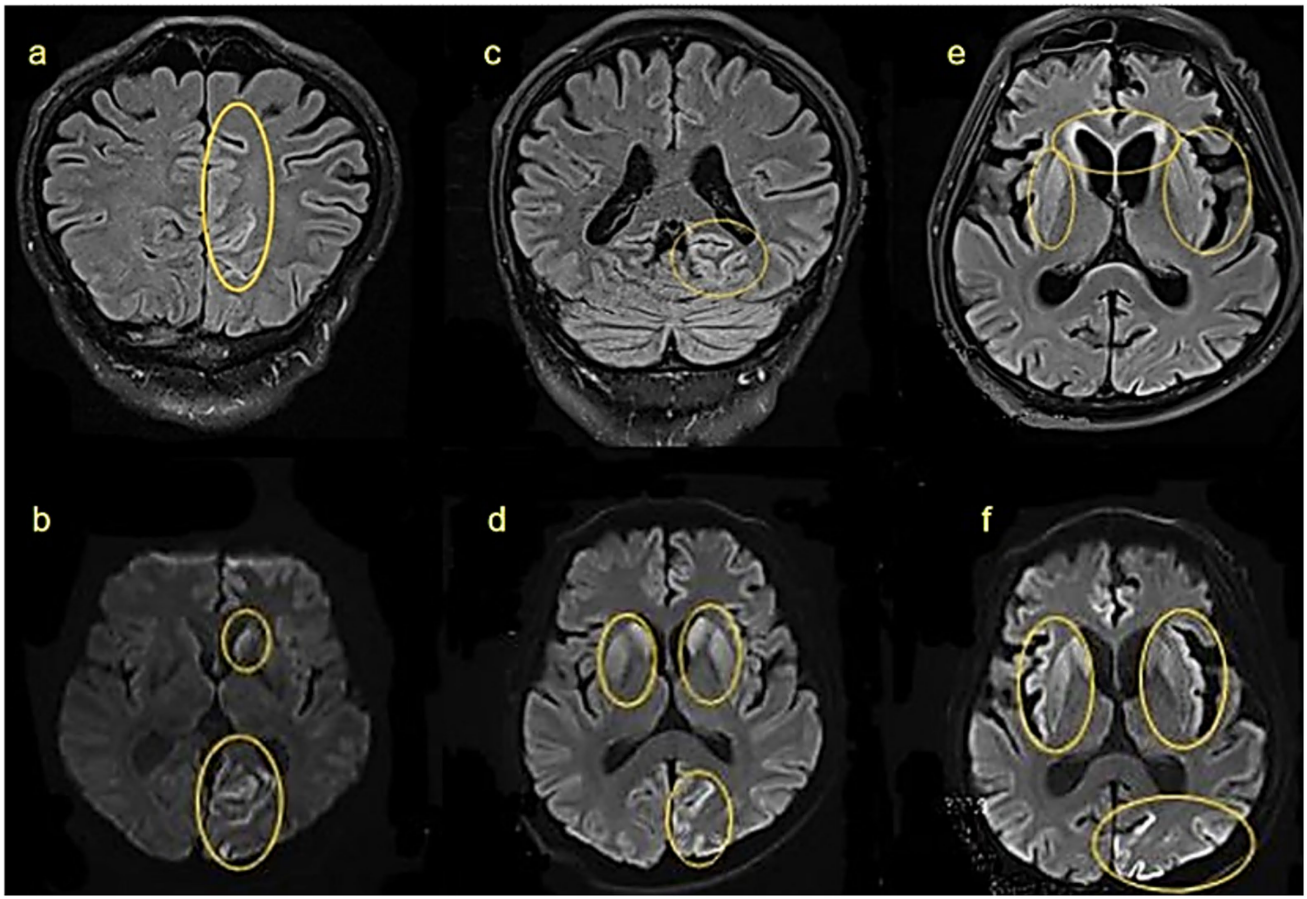
Cranial MRI findings at different stages of the same patient: **(a,b)** 8 weeks after onset: hyperintensities in the left caudate nucleus and left parieto-occipital cortex on FLAIR and DWI. **(c,d)** 12 weeks after onset: lesions extend bilaterally to caudate and lentiform nuclei. **(e,f)** 18 weeks after onset: further involvement of bilateral frontal lobes and insulae. Yellow circles highlight abnormal signals.

To illustrate the dynamic progression of sCJD, one patient underwent serial EEG and MRI examinations over 23 weeks after disease onset. EEG metrics and imaging findings are summarized below, and in Figures 2, 3.

The patient underwent the first EEG examination 8 weeks after the onset of the disease (Figure 2a). EEG showed a disorganized background with a slowed dominant rhythm (8 Hz mixed with 3–6 Hz slow waves) and emerging triphasic waves limited to the left frontal region. The triphasic waves appeared sporadically or intermittently in short to long durations, with initial periodicity. By week 9 post-onset, the second EEG examination (Figure 2b) showed sever background with widespread slow wave abnormalities (mainly irregular 2–7 Hz slow waves). Bilaterally and widespread PSWC appeared, repeating at intervals of 0.4–0.6 s. By week 11 post-onset, during the third EEG examination (Figure 2c), the patient’s disease deteriorated, and he entered in a coma. The background activity rhythm was slower than before, mainly 1.5–4 Hz slow waves. The interval of PSWC became longer at 0.8–1.4 s, and the spikes became rounded. By week 16 post-onset, during the fourth examination (Figure 2d), the background activity slow waves evolved into a rhythm of 1.5–3 Hz, the voltage was lower than before, and the period intervals of PSWC evolved into1–1.5 s. By week 23 post-onset (Figure 2e), EEG examination was performed in a coma. As the disease progressed, the EEG showed that PSWC were replaced by a burst-suppression pattern on a suppressed background, consistent with end-stage disease. In a short, during 8–16 weeks post-onset, the patient’s EEG showed PSWC from sporadically to periodically, and the interval gradually became longer. At 23 weeks of illness, the characteristics of PSWC disappeared.

Clinically, the patient’s clinical symptoms worsened, starting with memory impairment in the early stages and gradually progressing to lethargy, irritability, myoclonus, and finally coma.

MRI findings correlated closely with this progression. By 8 weeks post-onset, FLAIR and DWI hyperintensities were observed in the left caudate nucleus and left occipitoparietal cortex (Figures 3a,b). By 12 weeks post-onset, as the disease progressed, abnormalities had spread to bilateral caudate nuclei, putamen, and the left occipitoparietal region (Figures 3c,d), at 18 weeks post-onset, further extension was seen into the bilateral caudate nuclei, lentiform nuclei, bilateral frontal lobes, insular lobes, and the left occipital-parietal cortex (Figures 3e,f). These changes may be related to further neuronal damage and the deposition of prion proteins.

**Figure 4:**
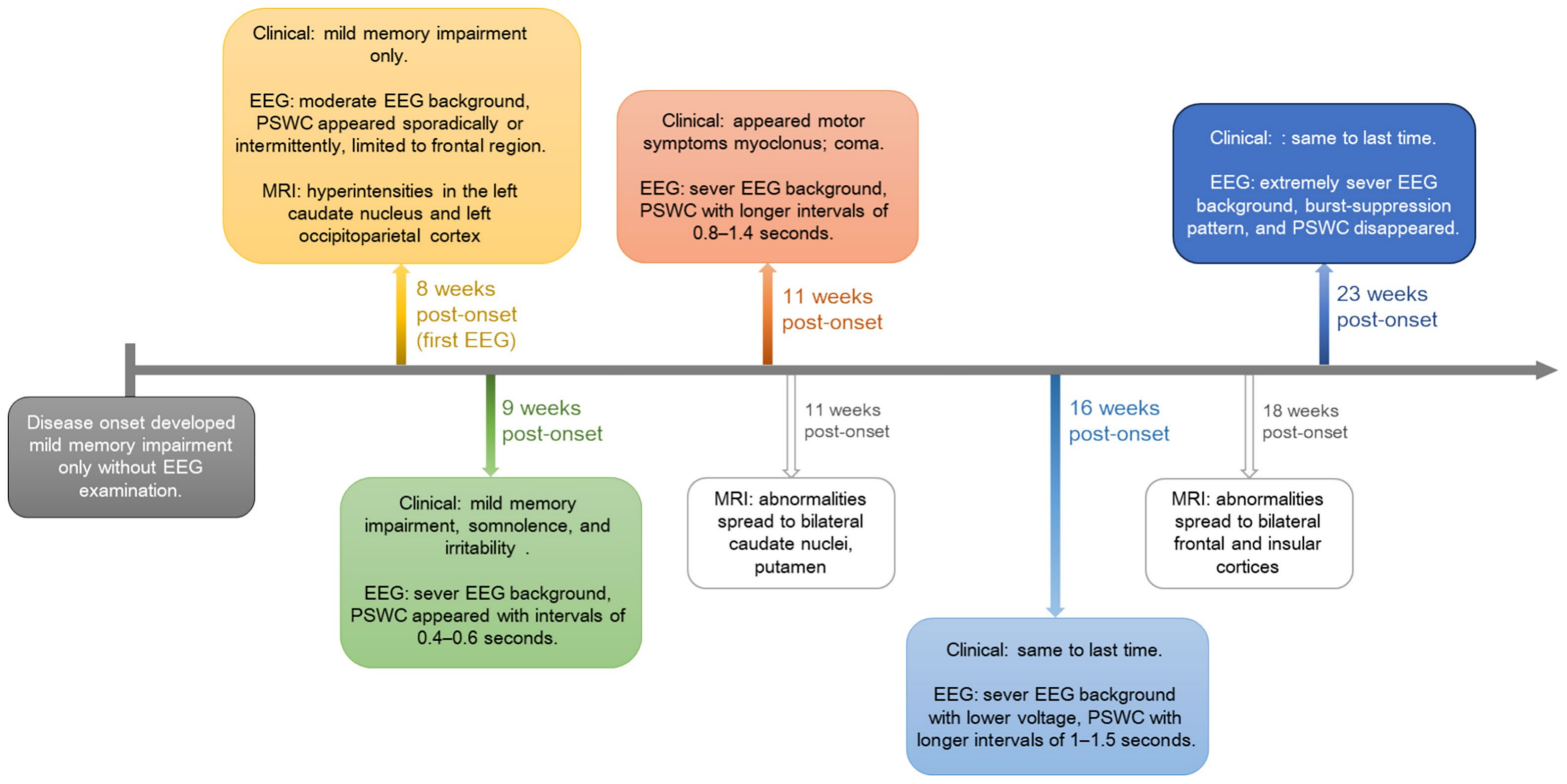
Visual timeline illustrating the longitudinally clinical, EEG, and MRI changes for the followed patient. PSWC, periodic sharp-wave complexes.

This longitudinal profile underscores the temporal evolution of electrophysiological and structural changes in sCJD, highlighting the value of combined EEG and MRI monitoring for tracking disease progression (Figure 4; Supplementary Table S1).

### 3.3 Results of statistical analyses

No significant correlations were observed between survival time and any two of age nor time of first EEG examination post-onset (all *p* > 0.05). Difference of survival time was not detected between 14-3-3 protein positive patients and 14-3-3 protein negative patients. Difference of survival time was also not detected between patients with typical PSWC and patients with atypical PSWC. Among patients with typical PSWC, 51.6% showed severe background activity, compared to 48.4% in those showed mild or moderate background activity. The difference was not statistically significant. Among patients examined within 4 weeks, 82.4% showed PSWC, compared to 85.7% in those examined after 4 weeks. The difference was not statistically significant. In addition, differences of survival time were not detected among patients with mild, moderate, nor sever background activities. Log-rank (Mantel-Cox) test showed that there were no differences in the median age of survival time for individuals with mild, moderate, or severe background activities (Figure 1b).

## 4 Discussion

This study revealed approximately 3% of overall survival rate in the 37 sCJD patients. The median survival time was about 3 months. The CSF 14–3-3 protein exhibited a notably high positivity rate of 80%. Nearly 50% of CJD patients underwent their first EEG examination within 1 month of symptom onset; over 80% presented typical PSWC; and about 90% grade at moderate to severe abnormalities in background rhythm. Follow-up on one patient demonstrated that as the disease progressed, the EEG initially displayed typical PSWC, which eventually faded. The patient’s basic EEG rhythm gradually slowed, background activity deteriorated, and the main regions of involvement expanded.

CJD is a fatal neurodegenerative disorder caused by the abnormal folding and accumulation of prion proteins (Watson et al., 2021). Consistently, our study revealed overall survival rate of 3% in our sCJD cohort. Clinical manifestations of CJD are diverse and often confused with other types of dementia, making diagnosis particularly challenging in the initial stages (Corriveau-Lecavalier et al., 2022). As the disease progresses, PSWC on EEG and positive 14-3-3 protein of CSF became a key diagnostic marker. This study showed that among 37 patients with sCJD, nearly 50% underwent their first EEG examination within 1 month of symptom onset, and over 80% of them already exhibited PSWC with moderate to severe background rhythm abnormalities, further confirming the central role of PSWC in the diagnosis of sCJD. Furthermore, the positivity rate of the 14-3-3 protein in CSF was 80.0% in our cohort, which aligns with its established role as a cornerstone biomarker for sCJD. The high concordance between PSWC on EEG and 14-3-3 protein positivity in our study reinforces the combined value of these paraclinical tests in supporting the clinical diagnosis, according to the World Health Organization’s (1998) diagnostic criteria, especially in atypical cases or when one modality is equivocal.

However, our statistical analysis revealed that none of the parameters we examined—including age, time to first EEG, CSF 14-3-3 protein status, the presence of typical PSWCs, and initial background activity severity—showed a significant correlation with survival time. The lack of prognostic value for these commonly assessed clinical and electrophysiological markers is a critical finding. It underscores the uniformly aggressive and relentless nature of sCJD, where the disease course may be largely independent of these initial presentation factors once the diagnosis is established. This suggests that the underlying prion pathology, potentially driven by molecular subtype-specific tropism and propagation kinetics, is the predominant determinant of survival, overwhelming the variability introduced by the timing of diagnosis or early symptomatic features (Ng et al., 2024; Muroga et al., 2023).

The appearance of PSWC exhibits significant time dependency and subtype heterogeneity (Li et al., 2023). In our cohort, 16.2% of patients did not show typical PSWC in first time EEG examination, a finding that resonates with the study by Ng et al., where only 33% of patients in a large autopsy-confirmed cohort exhibited PSWC on EEG (Ng et al., 2024). The stark difference in PSWC prevalence between studies (83% in ours vs. 33% in Ng’s) is likely attributable to the distribution of molecular subtypes. Ng’s study revealed a high proportion (38%) of VV1 and MM2 subtypes, which are notoriously associated with low PSWC positivity. The high PSWC occurrence rate in our sample might suggest a predominance of PSWC-high-expression subtypes such as MM1/MV1, which need further researches in the future. This is further supported by Muroga et al. (2023), who reported absent PSWC (0/12) in V180I genetic CJD, indicating that neuroimaging abnormalities can precede electrophysiological changes in certain subtypes, thereby diminishing PSWC sensitivity (Tschampa et al., 2005). Therefore, our findings, combined with existing literature, emphasize that EEG interpretation must be integrated with molecular subtyping and neuroimaging for accurate diagnosis, particularly for early cases with a “normal” EEG, where vigilance for atypical subtypes is paramount.

It is noteworthy that even in EEGs that appear “normal” on visual analysis, there may still be micro-abnormalities in functional connectivity (Harrington et al., 2021). The study by Pirker-Kees et al. (2025) pointed out that in early CJD patients, even without PSWC or significant slow waves, the global efficiency of the default mode network in the gamma band is significantly reduced. This indicates that in the very early stages, neural network integrity is already impaired before macro-electrical pathological patterns emerge. This concept of network desynchronization is consistent with the dynamic EEG evolution we observed in our longitudinal case, where PSWC appeared at week 8 and was replaced by burst-suppression by week 23, reflecting a pathological progression towards complete electrical silence.

Combining EEG-detected functional derailment with MRI-DWI-mapped structural injury creates a richer lens through which to decipher the evolving pathology of sCJD (Renzo et al., 2024). Our longitudinal follow-up demonstrated a tight synchrony between EEG changes (emergence/disappearance of PSWC, background slowing) and the anatomical expansion of MRI-DWI lesions from unilateral to bilateral involvement. This electro-clinical-imaging concordance highlights EEG’s sensitivity in reflecting disease severity and progression. While advanced techniques like 7 T MRI with integrated EEG nets can achieve millisecond-millimeter precision in healthy controls (Cicero et al., 2024), our study within the sCJD natural history shows that even conventional 3 T MRI and 10–20 EEG can reveal a consistent spatiotemporal coupling between lesion spread and electrophysiological deterioration, as also observed in a case by Ganesh et al. [11]. This reinforces that the calendar of sCJD can be tracked through both volts and voxels.

Notably, the early clinical manifestations of sCJD overlap with those of multiple rapidly progressive encephalopathies, and EEG features play a critical role in differential diagnosis. For autoimmune encephalitis such as anti-NMDAR encephalitis, the typical EEG finding is *δ* brushes against a background of diffuse slow waves, often accompanied by status epilepticus or focal discharges—these patterns differ significantly from the PSWC of sCJD (Xiao et al., 2024). In our cohort, 83.8% of patients exhibited typical PSWC, and no obvious post-epileptic discharge suppression, which can be distinguished from the non-periodic, variable discharge patterns of autoimmune encephalitis. For toxic-metabolic encephalopathies (e.g., hepatic encephalopathy, alcoholic encephalopathy) (Bögli et al., 2024), EEG abnormalities mainly present as diffuse *θ*/δ slow waves; some patients with hepatic encephalopathy may show triphasic waves (Kidokoro and Shiraki, 2025), but these waves are often accompanied by incomplete disappearance of background rhythm and can be reversed with the correction of metabolic disorders. In contrast, the PSWC of sCJD gradually worsen with disease progression, eventually evolving into burst-suppression or electrical silence—a process confirmed by the 23-week follow-up of one patient in this study, which stands in stark contrast to the reversible EEG changes of toxic-metabolic encephalopathy. In addition, for other rapidly progressive dementias (e.g., Lewy body dementia, frontotemporal dementia) (Rostamikia et al., 2024), EEG findings are mostly focal slow waves or mild background abnormalities, and typical PSWC rarely occur. In our study, no patients had no detectable PSWC, and about half of them presented PSWC within 4 weeks post-onset—this further indicates that PSWC has relatively high specificity for the differential diagnosis of sCJD.

This study has several limitations that should be acknowledged. First, its retrospective, single-center design with a relatively small sample size may introduce selection and recall biases, and limits the generalizability of our findings. Second, the lack of long-term follow-up and comprehensive end-stage data for all cohort restricts a full characterization of the disease’s terminal phase. Third, although all EEG analyses were conducted by experienced neurologists, the visual interpretation of EEG patterns, despite standardized criteria, retains a degree of subjectivity. Finally and most importantly, the absence of neuropathological confirmation in all cases and the lack of data on prion protein subtypes (e.g., MM1, VV2) or PRNP genotyping prevent a molecular-level explanation of the heterogeneity observed in our cohort, such as the wide range of survival times and the high prevalence of PSWC.

In conclusion, this study delineates the dynamic electrophysiological and neuroimaging trajectory of sCJD, underscoring the high diagnostic yield of early EEG, particularly the presence of PSWC, and its synergistic value with CSF 14–3-3 protein testing and MRI. Despite the uniformly poor prognosis and the lack of correlation between common baseline markers and survival, the detailed longitudinal mapping of disease progression provides a crucial reference for clinical monitoring. Our findings advocate for the integration of EEG into the initial diagnostic workup of rapidly progressive dementia. Future efforts should focus on prospective, multi-center studies that incorporate molecular subtyping and fluid biomarkers to construct a robust multimodal framework for achieving ultra-early diagnosis and personalized prognostic stratification in sCJD.

## Data Availability

The original contributions presented in the study are included in the article/Supplementary material, further inquiries can be directed to the corresponding author.
