# Supplementary material for: From early symptoms to EEG silence: tracking the neurodegenerative course of sporadic Creutzfeldt-Jakob disease
Source: Front Hum Neurosci. 2025 Oct 28;19:1652773. doi: 10.3389/fnhum.2025.1652773 (PMC12602467; doi:10.3389/fnhum.2025.1652773)
Supplement: Supplementary file 1 [file Data_Sheet_1.docx]

**Supplementary method**

**Diagnostic Criteria for Creutzfeldt-Jakob Disease**

**[Chinese Diagnosis Guidelines 2021]**

**A. Clinical Symptoms and Signs**
(1) Core clinical symptom: Rapidly progressive dementia
(2) Major clinical symptoms and signs: Cerebellar impairment, psychiatric symptoms, myoclonus, visual disturbances, extrapyramidal signs, pyramidal tract signs, akinetic mutism
(3) Atypical clinical symptoms: Speech impairment, dizziness, sleep disorders, autonomic dysfunction, limb numbness or weakness

**B. Paraclinical Features**
(1) Positive cerebrospinal fluid (CSF) or skin real-time quaking-induced conversion (RT-QuIC)
(2) Brain MRI showing hyperintensity on diffusion-weighted imaging (DWI) or fluid-attenuated inversion recovery (FLAIR) in at least two cortical regions (frontal, temporal, parietal, occipital) and/or basal ganglia (caudate/putamen)
(3) Electroencephalogram (EEG) showing periodic sharp wave complexes (PSWCs)
(4) Positive CSF 14-3-3 protein

**C. Definitive Features**
(1) Neuropathological examination demonstrating neuronal loss, gliosis, spongiform change, or PrP^Sc^ positive amyloid plaque deposition
(2) Presence of protease-resistant PrP^Sc^ confirmed by immunohistochemistry or Western blot
(3) Specific mutation in the *PRNP* gene

**D. Diagnostic Criteria**
**Probable sporadic CJD (sCJD)**
(1) Core clinical symptom + any two major clinical symptoms/signs
(2) Any clinical symptom/sign + one or more paraclinical features (items 2–4 under Section B)

*Must also meet the following: Disease course generally <2 years, and other etiologies (e.g., encephalitis, mitochondrial encephalopathy) have been ruled out through comprehensive evaluation.*

**Probable genetic CJD (gCJD)**
Meets criteria for probable sCJD (item 1 or 2) + positive family history

**Very Probable sCJD**
(1) Meets criteria for probable sCJD (item 1) + one or more paraclinical features (items 2–4 under Section B)
(2) Progressive neuropsychiatric symptoms + paraclinical feature 1

**Very Probable gCJD**
Meets criteria for probable sCJD (item 1 or 2) + positive family history

**Definite sCJD**
Meets criteria for probable/very probable sCJD + any one definitive feature (items 1–2 under Section C)

**Definite gCJD**
Meets criteria for probable/very probable sCJD or gCJD + definitive feature 3 (*PRNP* mutation)

**Note:** RT-QuIC: real-time quaking-induced conversion; DWI: diffusion-weighted imaging; FLAIR: fluid-attenuated inversion recovery; PrP^Sc^: disease-associated prion protein; *PRNP*: prion protein gene; sCJD: sporadic Creutzfeldt-Jakob disease; gCJD: genetic Creutzfeldt-Jakob disease.
