# Supplementary material for: From early symptoms to EEG silence: tracking the neurodegenerative course of sporadic Creutzfeldt-Jakob disease
Source: Front Hum Neurosci. 2025 Oct 28;19:1652773. doi: 10.3389/fnhum.2025.1652773 (PMC12602467; doi:10.3389/fnhum.2025.1652773)
Supplement: Supplementary file 2 [file Table_1.docx]

| **Table S1 Follow-up of clinical symptoms and EEG examination in a patient with sCJD.** | | | | | | | |
| --- | --- | --- | --- | --- | --- | --- | --- |
| No. of Follow-up | Time post-onset (weeks) | Main clinical manifestations | Basic rhythm of EEG (Hz) | Background activity grades | PSWC | Main area of PSWC^a^ | Low voltage EEG (< 2μV) |
| 1 | 8 | Memory impairment | 8 mixed with 3-6 | Moderate | Present | Frontal area | Absent |
| 2 | 9 | Memory impairment, somnolence, and irritability | 2-7 | Severe | Present | Full lead | Absent |
| 3 | 11 | Memory impairment, somnolence, myoclonus, and coma | 1.5-4 | Severe | Present | Full lead | Absent |
| 4 | 16 | Memory impairment, somnolence, myoclonus, and coma | 1.5-3 | Severe | Present | Full lead | Absent |
| 5 | 23 | Prolonged somnolence, myoclonus, and coma | 1-3 | Extremely severe | Absent | Full lead | Present |
| ^a^According to the international 10-20 system. | | | | | | | |
| Abbreviation: EEG = Electroencephalography; PSWC = periodic sharp wave complexes; sCJD = sporadic Creutzfeldt-Jakob disease. | | | | | | | |
